# A Rare Entity: Primary Pulmonary Meningioma

**DOI:** 10.5146/tjpath.2021.01535

**Published:** 2023-01-15

**Authors:** Aynur Baş, Elgün Valiyev, Nur Dilvin Özkan, İsmail Tombul, Selcen Yonat, Muhammet Sayan, İsmail Cüneyt Kurul

**Affiliations:** Basaksehir Cam and Sakura City Hospital, Department of Thoracic Surgery, Istanbul, Turkey; Gazi University, School of Medicine, Department of Thoracic Surgery, Ankara, Turkey; Gazi University, School of Medicine, Department of Pathology, Ankara, Turkey

**Dear Editor,**

Meningioma is the most common tumor of the central nervous system (CNS). Except for the CNS, it can be detected at a rate of 1-2% in the head and neck region. Primary pulmonary meningiomas (PPM) are extremely rare. They are detected incidentally as a solitary pulmonary nodule during radiological examinations. On computerized tomography (CT), the lesions are generally seen as a uniform and homogeneous mass. Although they are usually benign and the prognosis is excellent, they may be subject to misdiagnosis and overtreatment. (1–3). Here we have aimed to present a case of primary pulmonary meningioma that appeared as a solitary pulmonary nodule and to discuss its clinical and pathological features.

A 57-year-old male patient with cough had been on follow-up for five years. His medical history included 30 pack-year smoking history, hypertension, arrhythmia, and hypercholesterolemia. In 2015, a well-circumscribed lesion 10 mm in diameter had been detected in the subpleural area of the left lower lobe with thorax CT. There was no pathological uptake of 18F-FDG on PET-CT. The patient was put on radiological follow-up. There was no change in the nature of the nodule during the 3-year follow-up period and in the control thorax CT obtained in 2018, and the follow-up was continued with chest x-rays. During follow-up in January 2020, it was determined that the chest nodule had a similar appearance with the previous follow-ups, but there was a slight increase in density, and a newly developing nodule with a size of 7 mm had appeared in the neighboring parenchyma (Figure 1). Based on the available findings, a decision was made for surgery to enable histopathological confirmation. A frozen section was sent following wedge resection for both lesions in the lower left lobe. The operation was terminated when the lesion 11 mm in diameter was reported as pulmonary meningioma as a result of frozen section analysis. The other nodule was a parenchymal lymph node and the surgical margins were tumor free. In the final histopathological analysis, it was reported that the 8 mm nodule diagnosed with PPM was immunohistochemically positive for vimentin, EMA, and progesterone receptors. Psammoma bodies were seen but mitosis and necrosis were not observed (Figure 2, Figure 3). The patient was evaluated with MRI for possible central nervous system meningioma but no meningioma was detected. The patient was discharged without any complications in the postoperative period.

**Figure 1:**
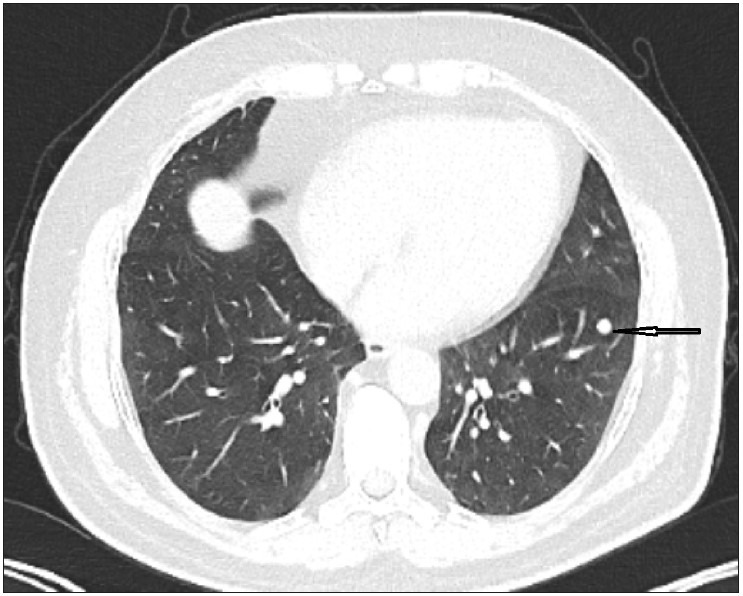
Thorax CT depicting a well-circumscribed lesion 10 cm in diameter in the subpleural area of the left lower lobe (arrow).

**Figure 2:**
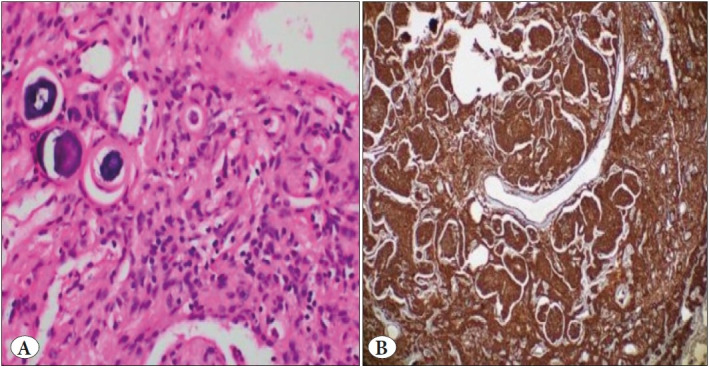
**A)** Microscopic examination showed spindle to round cells with whorl formation (H&E; x100). **B)** The tumor cells showed positive staining with vimentin (IHC; x100).

**Figure 3:**
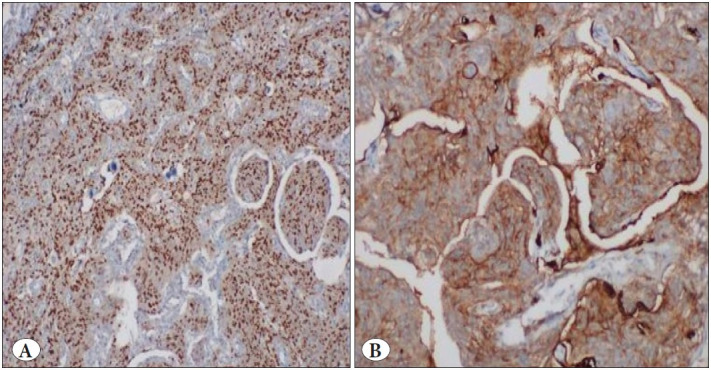
Immunohistochemical examination showed positive staining with **A)** progesterone receptor (IHC; x100) and **B)** EMA (IHC; x200).

PPMs are usually asymptomatic lesions that appear incidentally. However, patients with large tumors may have symptoms related to the localization, such as chest pain and cough. It has been reported that the lesion is generally detected between the ages of 40 and 60. Since the first case-report by Kemnitz in 1982, about 60 cases of PPM were published in the English literature (1). Our case was 57 years old, in accordance with the literature. Many benign PPMs have been reported to be misdiagnosed and consequently undergone extensive pulmonary resections and/or chemotherapy (4). In 2008, it was reported that 32% of patients who underwent major surgical resection and received chemotherapy had been misdiagnosed (4). In the literature, it is widely accepted that a wedge resection with tumor-free margins is sufficient for the treatment of benign PPM. The prognosis is reported to be excellent and extended resections are not required (3–5). In accordance with the literature, we performed wedge resection in the case. The definitive diagnosis of PPM was made histopathologically and immunohistochemically. Epithelial, transitional and fibrous types are possible. Immunohistochemically, vimentin and EMA are expressed together in most cases (3). Our patient also had a positive reaction for vimentin and EMA. The absence of necrosis and mitosis also supports the diagnosis of benign PPM. It has been reported that the prognosis of PPM is excellent and there was a case that has been followed for more than 20 years (5).

In conclusion, PPMs mostly appear radiologically in the form of pulmonary nodules, are usually benign, and have a good prognosis. PPM should also be kept in mind in the differential diagnosis of SPN.

## Conflict of Interest

The authors declare no conflicts of interest.
